# A novel risk score model based on gamma-aminobutyric acid signature predicts the survival prognosis of patients with breast cancer

**DOI:** 10.3389/fonc.2023.1108823

**Published:** 2023-03-08

**Authors:** Liping Yang, Jin Zhu, Lieliang Wang, Longbo He, Yi Gong, Qingfeng Luo

**Affiliations:** ^1^ Department of Breast Cancer Surgery, Jiangxi Cancer Hospital, Nanchang, China; ^2^ Department of Pathology, Jiangxi Cancer Hospital, Nanchang, China

**Keywords:** breast cancer, bioinformatics, ssGSEA, GABA, immune microenvironment, risk score

## Abstract

**Background:**

Gamma-aminobutyric acid (GABA) participates in the migration, differentiation, and proliferation of tumor cells. However, the GABA-related risk signature has never been investigated. Hence, we aimed to develop a reliable gene signature based on GABA pathways-related genes (GRGs) to predict the survival prognosis of breast cancer patients.

**Methods:**

GABA-related gene sets were acquired from the MSigDB database, while mRNA gene expression profiles and corresponding clinical data of breast cancer patients were downloaded from the Gene Expression Omnibus (GEO) and The Cancer Genome Atlas (TCGA) databases. Univariate Cox regression analysis was used to identify prognostic-associated GRGs. Subsequently, LASSO analysis was applied to establish a risk score model. We also constructed a clinical nomogram to perform the survival evaluation. Besides, ESTIMATE and ssGSEA algorithms were used to assess the immune cell infiltration among the risk score subgroups.

**Results:**

A GRGs-related risk score model was constructed in the TCGA cohort, and validated in the GSE21653 cohort. The risk score was significantly related to the overall survival of breast cancer patients, which could predict the survival prognosis of breast cancer patients independently of other clinical features. Breast cancer patients in the low-risk score group exhibited higher immune cell infiltration levels.

**Conclusion:**

A novel prognostic model containing five GRGs could accurately predict the survival prognosis and immune infiltration of breast cancer patients. Our findings provided a novel insight into investigating the immunoregulation roles of GRGs.

## Introduction

In women, breast cancer is more common than pulmonary cancer. Cancer statistics published by the ACS indicated that breast cancer will be the most common cancer in the United States. This trend will play out in China (1). Breast cancer is a primary threat to women’s health and it is the leading cause of cancer-associated death in women (2). At present, early detection and treatment of breast cancer can greatly decrease the mortality rate of breast cancer. However, it is often not diagnosed or discovered until after metastasis has happened (3). In addition, the main reason for the poor prognosis of breast cancer is that it starts from a local disease and spreads to other organs, which seriously hinders the effective treatment of breast cancer (4). A variety of treatments are currently available to treat breast cancer, including radiation therapy, hormone therapy, chemotherapy, and surgery. However, the prognosis for most breast cancer patients remains unfavorable, even those treated early (5, 6). These data demonstrated an urgent need to develop innovative approaches for breast cancer treatment to reduce its recurrence and death.

Gamma-aminobutyric acid (GABA) is non-protein amino widely found in vertebrates, plants, and microorganisms. The physiological effects of GABA are associated with preventing depression, promoting neuronal development, and regulating synaptic transmission (7, 8). In addition, various other pharmacological activities of GABA have also been reported, including intestinal protection, anti-allergy, anti-inflammatory, anti-cancer, etc. (9). GABA also contributes to the development and function of the immune system. According to a relevant study, GABA, as an immunomodulator, can regulate T cell proliferation and change T cell migration (10). Recent findings uncovered that tumor cell-derived GABA contributes to β-catenin-mediated immunosuppression and cancer cell growth (11). GABA derived from B cells recruits macrophages that generate IL-10 and suppress anti-tumor immunity (12). In addition, some studies have demonstrated that GABA receptors could inhibit cancer cell proliferation and suppress migration (13, 14). GABA type A receptor family genes exhibited distinct diagnostic and prognostic values for colon adenocarcinoma patients (15). Deficient GABA transaminase is associated with poor prognosis and cancer progression in hepatocellular carcinoma due to its role in tumor immunity (16). GABA level is a prognostic marker for breast cancer patients (17). Thus, an in-depth study of the role of GABA in cancer will facilitate the identification of novel prognostic markers and may provide more targets for immunotherapy and chemotherapy in breast cancer patients. However, there are very few studies on the GABA signaling pathway in breast cancer.

In the present study, we aimed to investigate the genes associated with the GABA signaling pathway to establish a novel prognostic risk model for breast cancer patients. The GABA signature was closely associated with the tumor immune microenvironment and can effectively predict the prognosis for breast cancer patients. In clinical diagnosis and treatment, the prognostic risk model constructed in the present study can help clinicians more accurately identify breast cancer patients with poor prognosis, and provide patients with more targeted examination and treatment. At the same time, our study also provides a theoretical basis and data support for further research on the role of GABA in breast cancer.

## Methods and materials

### Acquisition of data

For the discovery cohort, the transcriptome expression profiles and corresponding clinical data of breast cancer patients were downloaded from The Cancer Genome Atlas (TCGA). This discovery cohort contained 1109 breast cancer samples and 113 non-cancer samples. For the validation cohort, GSE21653 (252 breast cancer samples) dataset, with detailed clinical data, was downloaded from the Gene Expression Omnibus (GEO); the GSE22820 dataset (176 tumor samples and 10 normal samples) was also downloaded from GEO. Five GABA pathways-related datasets (WP GABA RECEPTOR SIGNALING, WP GABA METABOLISM AKA GHB, REACTOME GABA SYNTHESIS RELEASE REUPTAKE AND DEGRADATION, REACTOME GABA RECEPTOR ACTIVATION, BIOCARTA GABA PATHWAY) were acquired from MSigDB database (https://www.gsea-msigdb.org/). A total of 97 GABA pathways-related genes (GRGs) were selected for the research (**Table S1**). The “limma” package of R was applied to identify the differential expression of GRGs (DEGRGs) between the normal and tumor groups. The adjusted p-value was < 0.05, and the threshold was ∣logFC∣≥ 1. The volcano map of GRGs was generated using the “ggplot2” package of R.

### Construction of protein-protein interaction (PPI) network

The Search Tool for Retrieving Interacting Genes (STRING) (http://string-db.org/) was used to establish the PPI network, the results were visualized by Cytoscape software.

### Functional enrichment analysis of DEGRGs

The Kyoto Encyclopedia of Genes and Genomes (KEGG) and gene ontology (GO) enrichment analyses were performed to explore the potential biological functions of DEGRGs. The “clusterProfiler” package of R was used for enrichment analyses, and the significance threshold of p was < 0.05.

### Establishment of the prognostic risk score model based on DEGRGs

First, we performed a univariate Cox analysis to identify the prognosis-related DEGRGs in breast cancer patients. P < 0.05 was used as the screening condition. Subsequently, we used the “survival” and “glmnet” packages of R to carry out the least absolute shrinkage and selection operator (LASSO) Cox regression analysis. Then, the risk score for each sample was calculated according to the following formula: risk score = coefficient of Gene A *× expression of gene A +* coefficient of Gene B *× expression of gene B +…*coefficient of Gene N *× expression of gene N* (18). The breast patients were divided into low- and high-risk groups based on the median risk score. In addition, the Kaplan-Meier curve analysis was carried out to assess the difference in overall survival between low- and high-risk groups by using the “survival” package of R. The “timeROC” and “ggplot2” packages of R were used to assess the predictive ability of the risk score model.

### Establishment of nomogram

The “rms” and “survival” packages of R were used to construct a nomogram based on the risk score. An evaluation calibration curve was generated by plotting the nomogram-predicted survival probability and the observed survival probability.

### Assessment of immune cell infiltration between the two risk score groups

Estimation of STromal and Immune cells in MAlignant Tumours using Expression (ESTIMATE) is an algorithm that applies gene expression profiles to calculate the score of immune and stromal cells in cancer tissue (19). Single-sample gene set enrichment analysis (ssGSEA) was used to quantify the score of immune cells in the tumor samples. The “GSVA” package of R was applied to perform the ssGSEA (20). The results of ESTIMATE and ssGSEA were presented in the form of a histogram. In addition, the “ggplot2” package of R was used to evaluate the correlation between risk score and immune cells.

### Collection of samples and quantitative real-time polymerase chain reaction (qRT-PCR)

We collected 8 pairs of cancer tissues and peritumoral normal tissues from patients with breast cancer who received surgery between 2020 and 2021 at the Department of breast cancer surgery, Jiangxi Cancer Hospital. Before the surgery, patients did not undergo immunotherapy, chemotherapy, or radiotherapy. This study was approved by the ethics committee of Jiangxi Cancer Hospital. The RNA extraction reagent (ThermoFisher) was used to extract the total RNA from the tissue. The PrimeScript RT reagent kit (ThermoFisher) was used to perform the cDNA synthesis. Then, the Fast SYBR Green Master Mix (TaKaRa) was used to quantify the gene expression. The qRT-PCR was carried out on a StepOne Real-time PCR system (Applied Biosystems). The 2^–ΔΔCt^ method was applied to quantify the relative gene expression level. The primers were listed in **Table S2**.

### Tumor immune estimation resource (TIMER) database

The TIMER database (https://cistrome.shinyapps.io/timer/) is an online analysis tool, mainly used to visualize and analyze the level of tumor-infiltrating immune cells and calculate the abundance of various tumor immune cells from the TCGA database (21). In this study, we used the TIMER database to analyze the correlation between gene expression and immune cell infiltration in breast cancer.

## Results

### Analysis of DEGRGs in breast cancer

**Figure 1:**
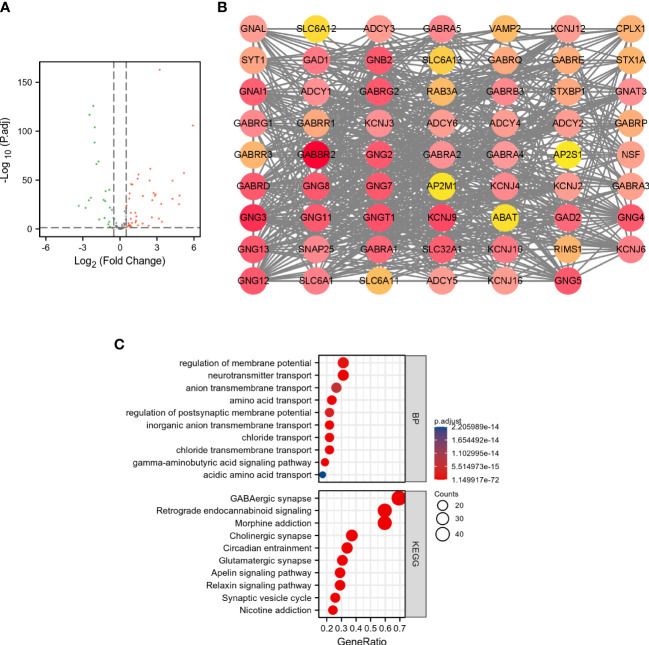
Analysis of DEGRGs in breast cancer. **(A)** Volcano plot of GRGs between tumor and normal groups. The green dots represent down-regulated genes, and the red dots represent up-regulated genes. **(B)** PPI network of DEGRGs. **(C)** Enrichment analyses of DEGRGs.

As shown in **Figures 1A, B**, a total of 43 DEGRGs were identified in the TCGA dataset, which included 28 up-regulated genes and 15 down-regulated genes. In addition, the results of GO-BP enrichment analysis indicated that these DEGRGs were significantly enriched in the regulation of membrane potential, neurotransmitter transport, anion transmembrane transport, amino acid transport, regulation of postsynaptic membrane potential, inorganic anion transmembrane transport, chloride transport, etc. The results of KEGG indicated that these DEGRGs were involved in GABAergic synapse, retrograde endocannabinoid signaling, morphine addiction, cholinergic synapse, circadian entrainment, glutamatergic synapse, apelin signaling pathway, relaxin signaling pathway, synaptic vesicle cycle, etc (**Figure 1C**).

### Construction and validation of the DEGRGs-related prognostic risk model

**Figure 2:**
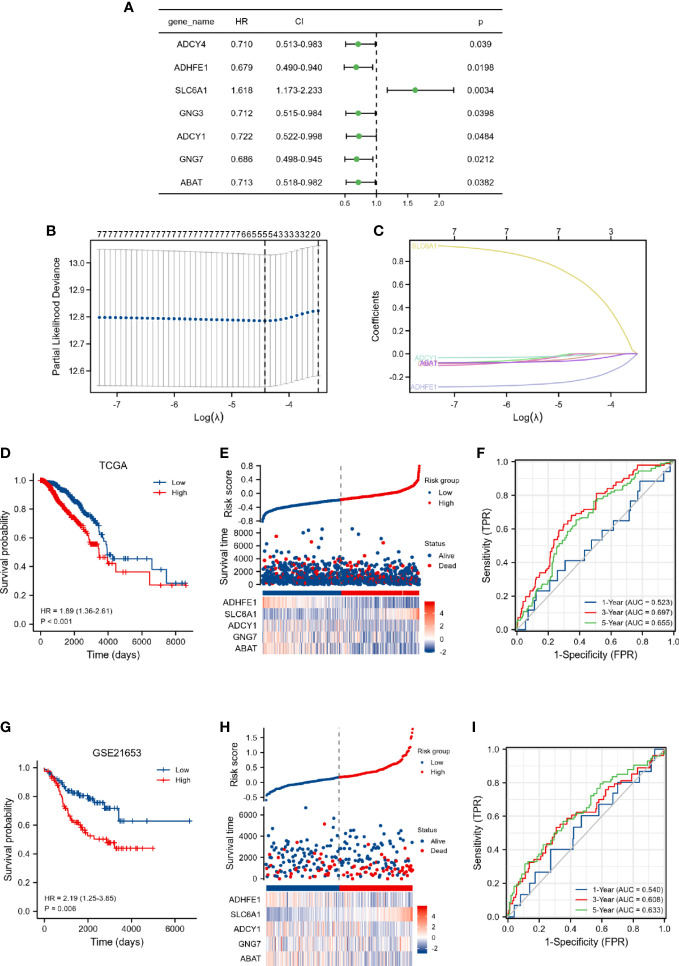
Construction of DEGRGs-related risk score model in TCGA dataset. **(A)** Univariate Cox regression analysis of DEGRGs. **(B, C)** LASSO regression analysis of 7 overall survival-related DEGRGs. **(D)** Kaplan-Meier survival analysis of risk score in the TCGA. **(E)** The expression level of DEGRGs (below), survival status (middle), and the distribution of risk scores in the low- and high-risk score groups (upper). **(F)** Time-dependent ROC curve analyses. Validation of DEGRGs-related risk score model in GSE21653 dataset. **(G)** Kaplan-Meier survival analysis of risk score in the GSE21653. **(H)** The expression level of DEGRGs (below), survival status (middle), and the distribution of risk scores in the low- and high-risk score groups (upper). **(I)** Time-dependent ROC curve analyses.

As shown in **Figure 2A**, 7 overall survival-related genes were identified by univariate Cox regression analysis of the 43 DEGRGs. Subsequently, the 7 overall survival-related genes were subjected to the LASSO Cox regression analysis, and 5 genes were identified and selected for the construction of the prognostic risk model (**Figures 2B, C**). The risk score model was calculated by the following formula: risk score = 0.5792 *× expression of* SLC6A1 *+* (-0.0485) *× expression of* ABAT + (-0.006) *×* expression of ADCY1 + (-0.2103) *×* expression of ADHFE1 + (-0.0188) *× expression of* GNG7. The results of Kaplan-Meier curves indicated that breast cancer patients in the high-risk score group had a worse prognosis than those in the low-risk group (**Figure 2D**, p < 0.001). **Figure 2E** presented the survival status of breast cancer patients and the expression of risk score-related genes sorted by risk score, and results indicated that patients in the high-risk score group were more likely to die. In addition, we used the ROC curve to assess the predictive performance of the risk score model. The AUC for the risk score model was 0.523 at 1-year, 0.697 at 3-year, and 0.655 at 5-year (**Figure 2F**), these results indicated that the risk score model exhibited discrimination.

Furthermore, the prognostic values of the risk score and clinical characteristics were further investigated by univariate and multivariate analyses. As presented in **Table S3**, our results indicated that age, M stage, and risk score were independently associated with the outcome in breast cancer patients.

We also validated the predictive ability of the risk score model by the GSE21653 dataset. Kaplan-Meier curves indicated that breast cancer patients in the high-risk score group had a worse prognosis (**Figure 2G**, p = 0.006). The breast cancer patients in the high-risk score group were more likely to die (**Figure 2H**). The AUC for the risk score model was 0.54 at 1-year, 0.608 at 3-year, and 0.633 at 5-year (**Figure 2I**). The findings of the discovery cohort and validation cohort were comparable, implying that our prognostic model has considerable stability for the prediction of breast cancer patients.

### Construction of the nomogram

**Figure 3:**
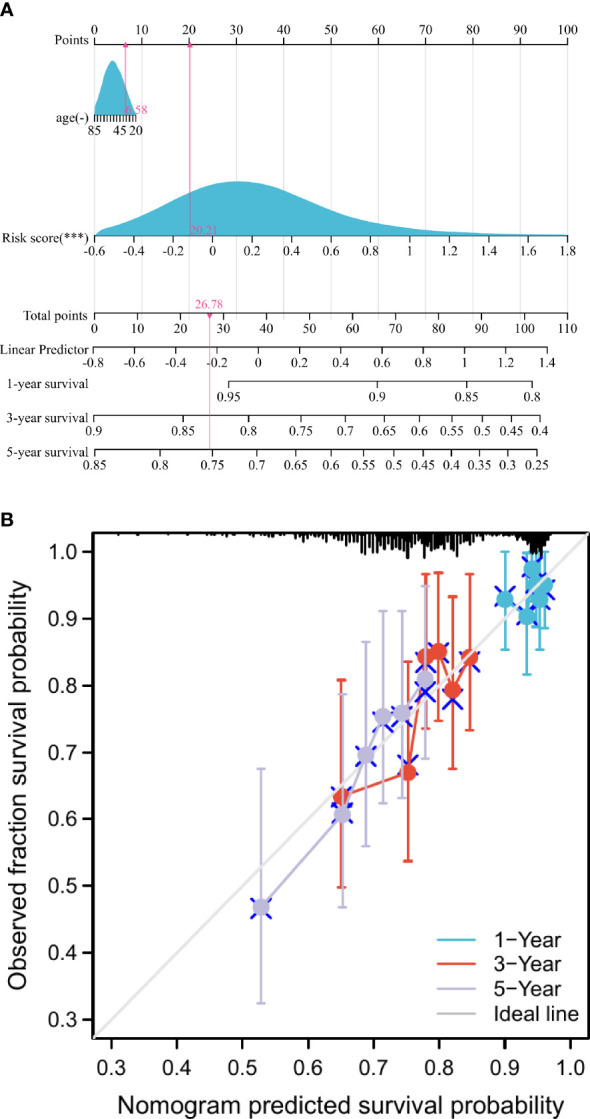
Construction and assessment of the nomogram. **(A)** Development of nomograms for predicting prognosis. **(B)** Calibration curve for predicting 1-, 3-, and 5-year survival in breast cancer patients. ***p < 0.001.

The 1-, 3-, and 5-year prediction nomograms of breast cancer patients were drawn based on the risk score (**Figure 3A**). In addition, as shown in **Figure 3B**, the 1-, 3-, and 5-year calibration curves for overall survival showed a good fit between observed and predicted survival, the findings also demonstrated the accuracy of the prognostic nomogram.

### Differences in immune status between low- and high-risk score groups

**Figure 4:**
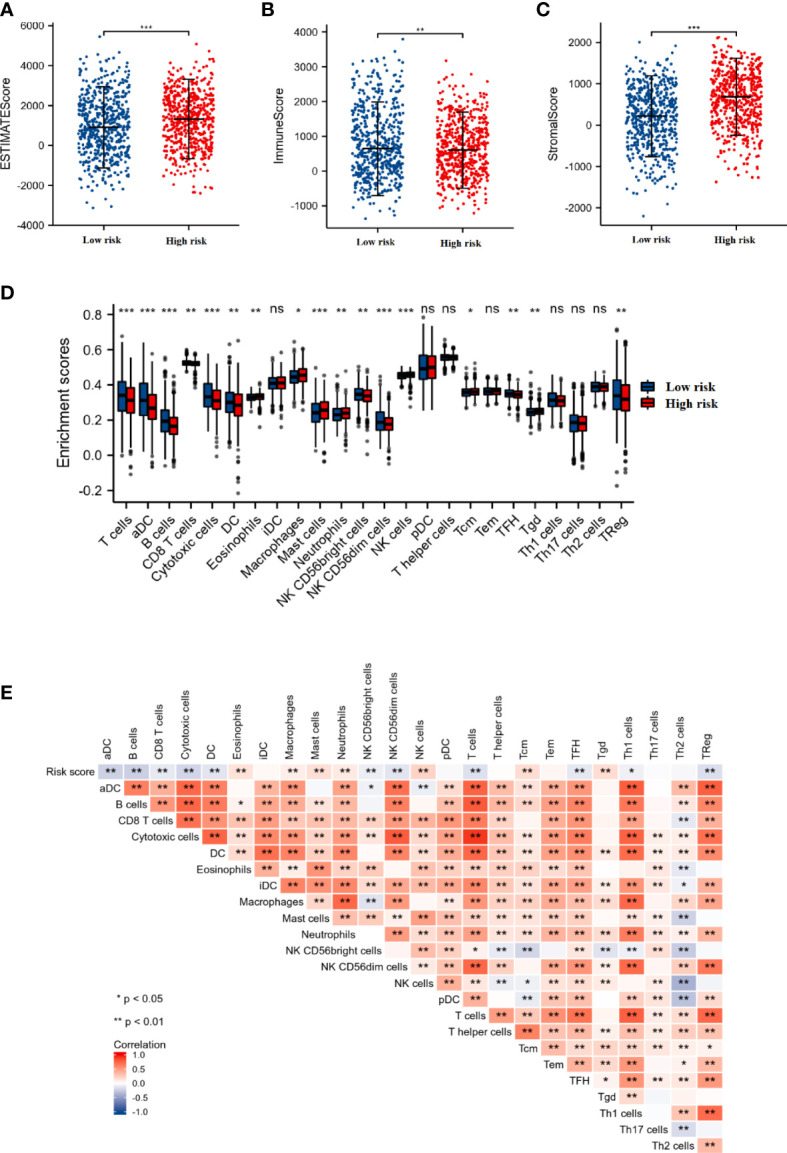
The association between the immune status and risk score. The comparison of ESTIMATE score **(A)**, Immune score **(B)**, and Stromal score **(C)** between the low- and high-risk score groups. **(D)** The box plot presented the differences in immune cell infiltration between the low- and high-risk score groups. **(E)** Correlation analysis of the risk score and immune cell infiltration in breast cancer. *p < 0.05, **p < 0.01, ***p < 0.001. "ns" is no significant difference.

We also investigated whether the tumor microenvironment was related to the risk score. As shown in **Figures 4A–C**, the high-risk group had a significantly higher ESTIMATE score (p < 0.001), higher stromal score (p < 0.001), and lower immune score (p < 0.01) compared to those in the low-risk score group. In addition, the results of ssGSEA indicated that the enrichment scores of T cells, aDC (activated dendritic cells), B cells, CD8 T cells, cytotoxic cells, DC (dendritic cells), NK CD56bright cells, NKCD56dim cells, TFH, and TReg in the high-risk score group were significantly lower than those in the low-risk score group (p < 0.05). Whereas, the enrichment scores of eosinophils, macrophages, mast cells, neutrophils, NK cells (natural killer cells), Tcm, and Tgd in the high-risk score group were significantly higher than those in the low-risk score group (p < 0.05) (**Figure 4D**).

Furthermore, we also performed the correlation analysis between the DEGRGs-related risk score and immune cell infiltration. As shown in **Figure 4E**, the infiltration level of the T cells, aDC, B cells, CD8 T cells, cytotoxic cells, DC, NK CD56bright cells, NKCD56dim cells, TFH, and TReg were negatively associated with the risk score, while the levels of the eosinophils, macrophages, mast cells, neutrophils, NK cells, Tcm, and Tgd were positively associated with the risk score.

### Evaluation of the prognostic value of DEGRGs in the TCGA dataset

**Figure 5:**
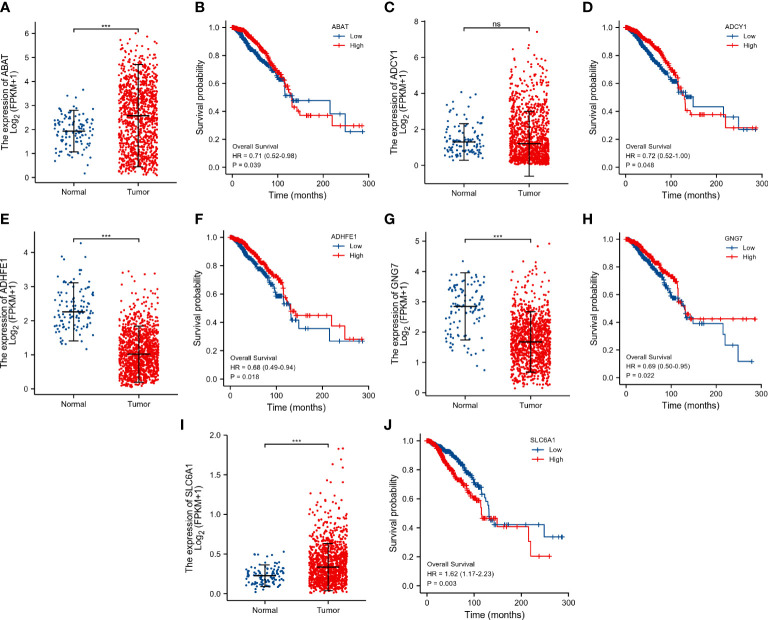
Evaluation of the prognostic value of DEGRGs in the TCGA dataset. The expression level of ABAT **(A)**, ADCY1 **(C)**, ADHFE1 **(E)**, GNG7 **(G)**, and SLC6A1 **(I)** in the TCGA dataset. Kaplan-Meier survival analysis of ABAT **(B)**, ADCY1 **(D)**, ADHFE1 **(F)**, GNG7 **(H)**, and SLC6A1 **(J)** in the TCGA breast cancer patients. ***p < 0.001. "ns" is no significant difference.

The expression level of ABAT (**Figure 5A**), and SLC6A1 (**Figure 5I**) was significantly up-regulated in the tumor group. In contrast, the expression level of ADHFE1 (**Figure 5E**) and GNG7 (**Figure 5G**) was significantly down-regulated in the tumor group. The up-regulation of ABAT (**Figure 5B**), ADCY1 (**Figure 5D**), and SLC6A1 (**Figure 5J**) were associated with poor prognosis in breast cancer patients (p < 0.05). The down-regulation of ADHFE1 (**Figure 5F**), and GNG7 (**Figure 5H**) was associated with poor prognosis in breast cancer patients (p < 0.05). There was no significant difference in the mRNA expression of ADCY1 (**Figure 5C**) in the primary breast tumor and normal breast tissue. These results indicated that ABAT, SLC6A1, ADHFE1, and GNG7 were the potential prognostic biomarkers for breast cancer.

### Validation of the expression level of the DEGRGs

**Figure 6:**
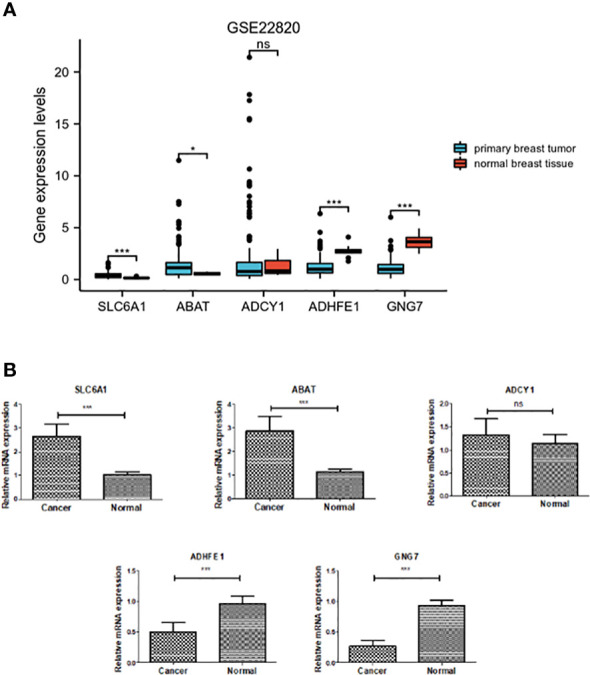
Verification of expression of the five DEGRGs. **(A)** Gene expression level of five DEGRGs in the GSE22820 dataset. **(B)** Gene expression level of five DEGRGs in 8 pairs of breast cancer tissue samples. *p < 0.05, ***p < 0.001. "ns" is no significant difference.

As shown in **Figure 6**, the expression level of DEGRGs was verified using the GSE22820 dataset and clinical samples. The mRNA expression level of ADHFE1 and GNG7 was significantly down-regulated in the primary breast tumor. In contrast, the expression level of ABAT and SLC6A1 was significantly up-regulated in the primary breast tumor. There was no significant difference in the mRNA expression of ADCY1 in the primary breast tumor and normal breast tissue. This result was consistent with the above finding.

### Correlation analysis of the DEGRGs and immune cell infiltration in breast cancer

**Figure 7:**
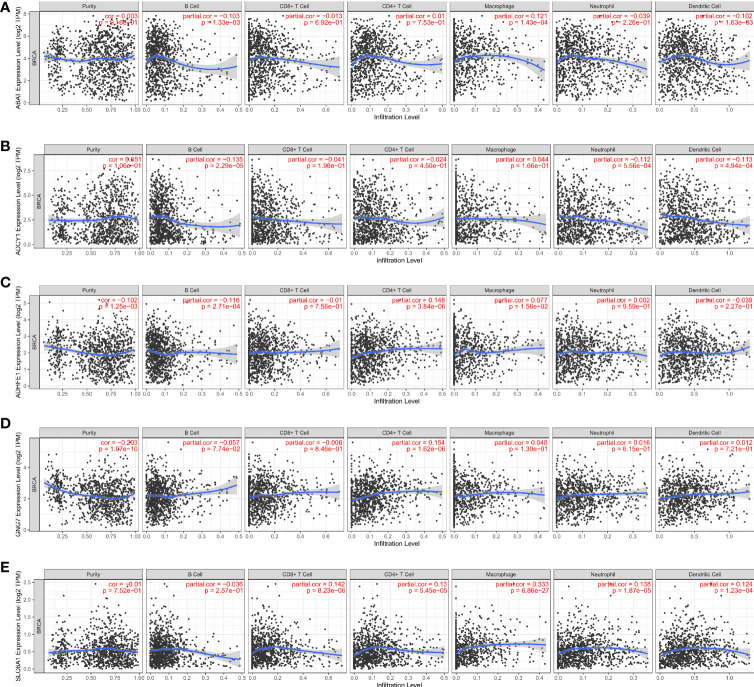
Correlation analysis of the ABAT **(A)**, ADCY1 **(B)**, ADHFE1 **(C)**, GNG7 **(D)** SLC6A1 **(E)** and immune cell infiltration levels in breast cancer.

As shown in **Figure 7A**, the ABAT expression had a significantly negative correlation with infiltrating level of B cell (p = 1.33×10^-3^), and dendritic cell (p = 1.63×10^-3^); the ABAT expression had a significantly positive correlation with infiltrating level of macrophage (p = 1.43×10^-4^). ADCY1 expression had a significantly negative correlation with infiltrating level of B cell (p = 2.29×10^-5^), neutrophil (p = 5.56×10^-4^), and dendritic cell (p = 4.94×10^-4^) (**Figure 7B**). ADHFE1 expression had a significantly negative correlation with infiltrating level of B cell (p = 2.71×10^-4^); ADHFE1 expression had a significantly positive correlation with infiltrating level of CD4+ T cell (p = 3.84×10^-6^) (**Figure 7C**). GNG7 expression had a significantly positive correlation with infiltrating level of CD4+ T cell (p = 1.62×10^-6^) (**Figure 7D**). SLC6A1 expression had significantly positive correlation with infiltrating level of CD8+ T cell (p = 8.23×10^-6^), CD4+ T cell (p = 5.45×10^-5^), macrophage (p = 6.86×10^-27^), neutrophil (p = 1.87×10^-5^), and dendritic cell (p = 1.23×10^-4^) (**Figure 7E**). Our findings indicated that these DEGRGs play a vital role in the immune infiltration of breast cancer.

## Discussion

Breast cancer is a typical heterogeneous cancer. It is the malignant tumor with the highest incidence rate in women and poses a serious threat to women’s health. Despite significant advances in the treatment of breast cancer in recent years. However, it is still very important to develop effective markers for the diagnosis and prognosis of breast cancer, especially in the early diagnostic detection of breast cancer (22). In our study, we intended to construct an efficient prognostic model for breast cancer patients and identify specific targets for the diagnosis and treatment of breast cancer.

In the present study, a DEGRGs-related risk score model that efficaciously classified breast cancer patients and predicted overall survival was constructed based on the GABA pathways. This risk score model comprised five genes (ABAT, SLC6A1, ADCY1, ADHFE1, and GNG7), and it had excellent predictive power in the discovery and validation cohorts. In addition, in terms of AUC for the risk model’s ROC curve, the prognostic model exhibited good diagnostic performance for the prediction of 3-year and 5-year survival rates. Multivariate analysis revealed that risk score was an independent prognostic factor for breast cancer patients. These findings implied that the risk model had significant clinical implications in the diagnosis and prediction of clinical outcomes of breast cancer patients. Furthermore, we also performed ESTIMATE and ssGSEA analyses to investigate the immune cell infiltration landscape between the low- and high-risk groups. Our findings revealed that the high-risk group exhibited a bad prognosis and an immunosuppressive microenvironment characterized by low immune score and low infiltration level of immune cells. Especially, in the high-risk group with poor prognosis, there was a significant decrease in the infiltration of CD8 T cells and dendritic cells (DC). The results were consistent with previous studies: a low CD8 T cells score was associated with poorer survival in triple-negative breast cancer (23); Breast cancer patients with low DC count tended to have shorter progression-free survival than patients with high infiltrated DC (24). These findings further showed that GRGs may play a vital role in the changed tumor immune microenvironment in breast cancer.

The proposed risk score model contained five GRGs, including ABAT, SLC6A1, ADCY1, ADHFE1, and GNG7. Gamma-aminobutyrate aminotransferase (ABAT) was down-regulated in liver cancer tissue and cell levels, and low expression of ABAT was associated with poor prognosis in liver cancer (25). In addition, the down-regulation of ABAT expression was associated with poor first-line endocrine therapy outcomes in patients with advanced disease (26). Solute carrier family 6 member 1 (SLC6A1) overexpression promoted clear cell renal cell carcinoma cell invasion, migration, and proliferation (27). SLC6A1 overexpression was associated with tumor progression and poor prognosis in patients with prostate cancer (28). SLC6A1 was up-regulated in colorectal cancer and can be used as an independent marker for colorectal cancer prognosis (29). Adenylyl cyclase 1 (ADCY1) overexpression inhibited glioma cell invasion, migration, and proliferation (30). Low expression of ADCY1 was related to pancreatic adenocarcinoma patients’ overall survival, and it was an independent risk factor for pancreatic adenocarcinoma (31). Alcohol dehydrogenase iron containing 1 (ADHFE1) encoded hydroxy acid-oxyacid trans-hydro enzymes involved in a variety of biological processes, including cancer (32). Up-regulation of ADHFE1 suppressed the proliferation of colorectal cancer cells through regulation of the cell cycle (33). ADHFE1 contributed to metabolic reprogramming with a reductive glutamine metabolism in breast tumors (34, 35). G Protein Subunit Gamma 7 (GNG7) is involved in inducing apoptosis and regulating cell proliferation (36, 37). GNG7 prevented tumorigenesis in lung adenocarcinoma cells by suppressing E2F transcription factor 1 (38). GNG7 was a promising prognostic biomarker and was related to immune cell infiltration in colorectal cancer (39). Overall, these GRGs were closely associated with tumor progression, which merits further investigation.

However, our study has some shortcomings. Although the expression level of five DEGRGs was validated by qRT-PCR in 8 pairs of clinical samples, a larger number of samples would make the results more reliable. In addition, the prognostic performance of the risk model needs to be validated in larger breast cancer cohorts.

## Conclusion

In conclusion, this was the first research to investigate the role of GABA-related pathways and construct the DEGRGs-related risk score model in breast cancer. This risk score model could effectively predict the prognosis of breast cancer patients. In addition, five DEGRGs associated with this risk model may be involved in the progression of breast cancer *via* impacting immune cell infiltration. Our findings could provide a scientific basis for the study of the GABA signaling pathway in breast cancer.

## Data availability statement

The datasets presented in this study can be found in online repositories. The names of the repository/repositories and accession number(s) can be found in the article/**Supplementary Material**.

## Author contributions

LY wrote the manuscript. JZ designed the study. LW, LH, and YG analyzed the data. QL reviewed and edited the manuscript. All authors contributed to the article and approved the submitted version.

## Supplementary material

Supplementary Table 1 GABA pathways-related genes (GRGs) were acquired from MSigDB database (https://www.gsea-msigdb.org/).
