# Supplementary figures and images for: A novel risk score model based on gamma-aminobutyric acid signature predicts the survival prognosis of patients with breast cancer
Source: Front Oncol. 2023 Mar 8;13:1108823. doi: 10.3389/fonc.2023.1108823 (PMC10031029; doi:10.3389/fonc.2023.1108823)

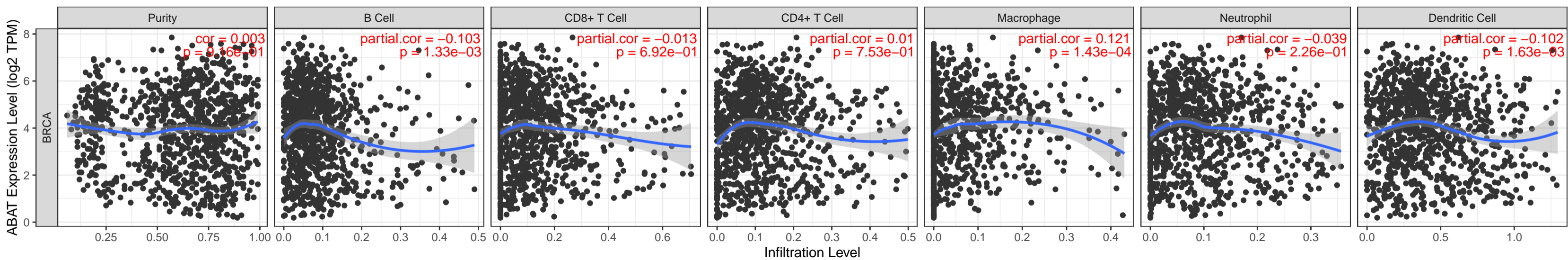

Supplement: Supplementary file 1 [file DataSheet_1.zip › raw data/Fig 7/Fig 7a.pdf]

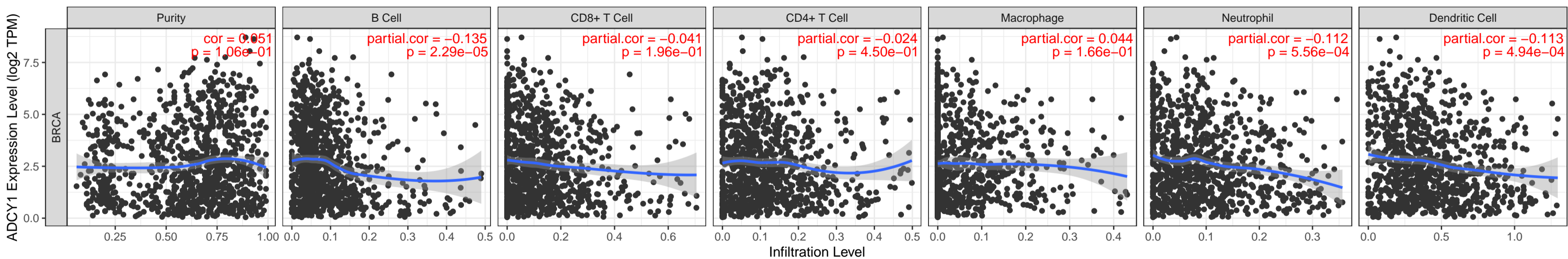

Supplement: Supplementary file 1 [file DataSheet_1.zip › raw data/Fig 7/Fig 7b.pdf]

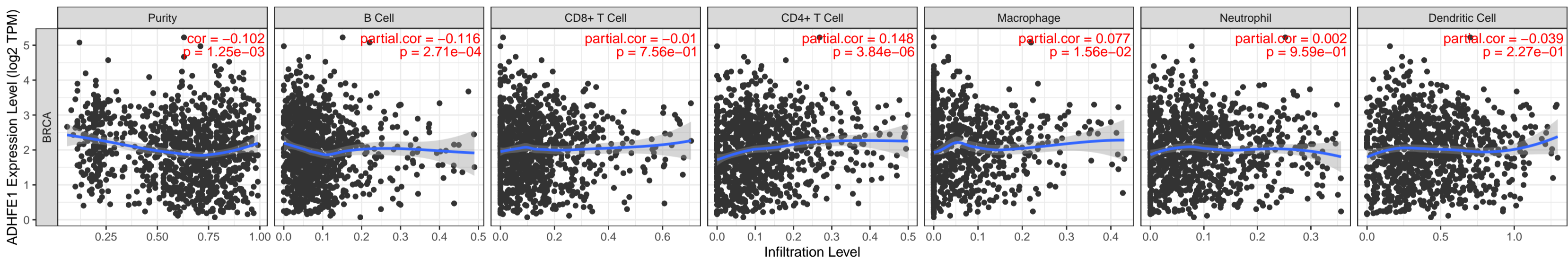

Supplement: Supplementary file 1 [file DataSheet_1.zip › raw data/Fig 7/Fig 7c.pdf]

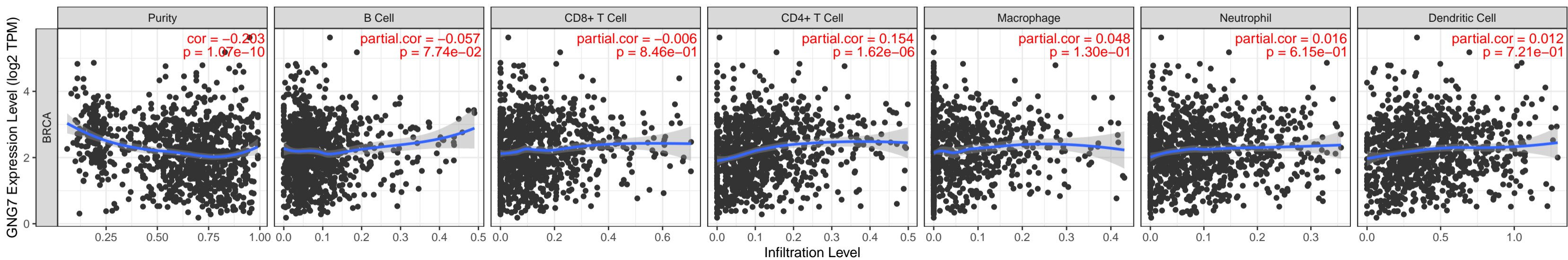

Supplement: Supplementary file 1 [file DataSheet_1.zip › raw data/Fig 7/Fig 7d.pdf]

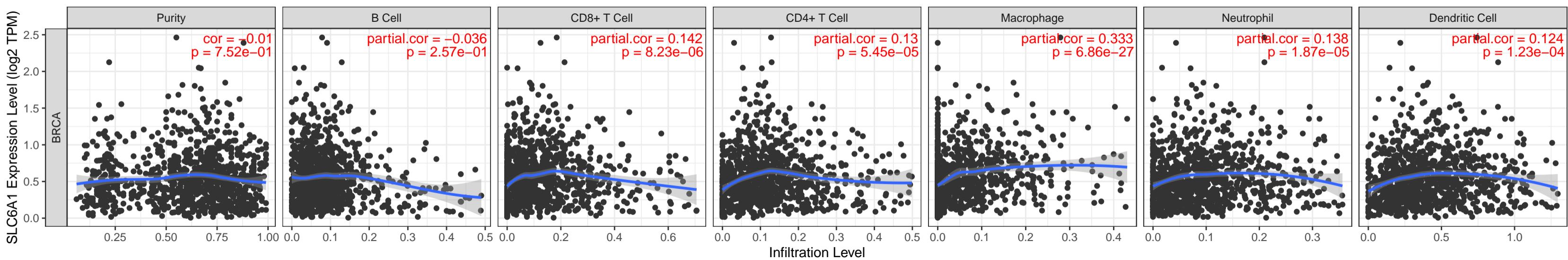

Supplement: Supplementary file 1 [file DataSheet_1.zip › raw data/Fig 7/Fig 7e.pdf]
