# Supplementary material for: A novel risk score model based on gamma-aminobutyric acid signature predicts the survival prognosis of patients with breast cancer
Source: Front Oncol. 2023 Mar 8;13:1108823. doi: 10.3389/fonc.2023.1108823 (PMC10031029; doi:10.3389/fonc.2023.1108823)
Supplement: Supplementary file 3 [file Table_2.docx]

Table S2 Sequences of primers used quantitative real-time PCR.

| Gene | Forward primer (5' to 3') | Reverse primer (5' to 3') |
| --- | --- | --- |
| ADHFE1 | AGGCTGCTAATCTGTATGCATC | CAGTAGTTTCACTCCCGGTT |
| GNG7 | TGGTGGAACAGCTACGCATAGA | CGGGCATGTTGCTCACAGTAG |
| ABAT | CCGACTACAGCATCCTCTCC | GGTTCTCTTTCACAAACTCTTCC |
| SLC6A1 | GTGAGGCAACTCCAAGGT | CAGATGGACGTGCGATGT |
| ADCY1 | GTCATGGAGGCTGCTGGCT | GATGGGATGGCACAATAAAGAAG |
| β-ACTIN | ACCACAGTCCATGCCATCAC | TCCACCACCCTGTTGCTGTA |
