# Supplementary material for: A novel risk score model based on gamma-aminobutyric acid signature predicts the survival prognosis of patients with breast cancer
Source: Front Oncol. 2023 Mar 8;13:1108823. doi: 10.3389/fonc.2023.1108823 (PMC10031029; doi:10.3389/fonc.2023.1108823)
Supplement: Supplementary file 4 [file Table_3.docx]

**Table S3** Univariate and multivariate analyses of clinical features and risk score in the breast cancer patients.

| Characteristics | Total(N) | Univariate analysis | |  | Multivariate analysis | |
| --- | --- | --- | --- | --- | --- | --- |
|  |  | Hazard ratio (95% CI) | P value |  | Hazard ratio (95% CI) | P value |
| T stage | 1079 |  |  |  |  |  |
| T1 | 276 | Reference |  |  |  |  |
| T2 | 629 | 1.332 (0.887-1.999) | 0.166 |  | 0.850 (0.424-1.703) | 0.646 |
| T3&T4 | 174 | 1.953 (1.221-3.123) | **0.005** |  | 0.846 (0.351-2.039) | 0.709 |
| N stage | 1063 |  |  |  |  |  |
| N0 | 514 | Reference |  |  |  |  |
| N1 | 357 | 1.956 (1.329-2.879) | **<0.001** |  | 1.284 (0.737-2.236) | 0.378 |
| N2 | 116 | 2.519 (1.482-4.281) | **<0.001** |  | 1.326 (0.497-3.541) | 0.573 |
| N3 | 76 | 4.188 (2.316-7.574) | **<0.001** |  | 1.724 (0.672-4.421) | 0.257 |
| M stage | 922 |  |  |  |  |  |
| M0 | 902 | Reference |  |  |  |  |
| M1 | 20 | 4.254 (2.468-7.334) | **<0.001** |  | 7.554 (1.800-31.705) | **0.006** |
| Pathologic stage | 1059 |  |  |  |  |  |
| Stage I | 180 | Reference |  |  |  |  |
| Stage II | 619 | 1.697 (0.985-2.922) | 0.057 |  | 1.745 (0.666-4.571) | 0.257 |
| Stage III | 242 | 2.962 (1.664-5.273) | **<0.001** |  | 3.449 (0.920-12.938) | 0.066 |
| Stage IV | 18 | 11.607 (5.569-24.190) | **<0.001** |  |  |  |
| Age | 1082 |  |  |  |  |  |
| <=60 | 601 | Reference |  |  |  |  |
| >60 | 481 | 2.020 (1.465-2.784) | **<0.001** |  | 2.329 (1.558-3.481) | **<0.001** |
| Histological type | 977 |  |  |  |  |  |
| Infiltrating Ductal Carcinoma | 772 | Reference |  |  |  |  |
| Infiltrating Lobular Carcinoma | 205 | 0.827 (0.526-1.299) | 0.410 |  |  |  |
| PR status | 1029 |  |  |  |  |  |
| Negative | 342 | Reference |  |  |  |  |
| Positive | 687 | 0.732 (0.523-1.024) | 0.068 |  | 0.857 (0.456-1.609) | 0.630 |
| Race | 993 |  |  |  |  |  |
| Asian | 60 | Reference |  |  |  |  |
| Black or African American | 180 | 1.525 (0.463-5.024) | 0.488 |  |  |  |
| White | 753 | 1.325 (0.420-4.186) | 0.631 |  |  |  |
| ER status | 1032 |  |  |  |  |  |
| Negative | 240 | Reference |  |  |  |  |
| Positive | 792 | 0.712 (0.495-1.023) | 0.066 |  | 0.630 (0.304-1.305) | 0.214 |
| PAM50 | 1042 |  |  |  |  |  |
| LumA | 561 | Reference |  |  |  |  |
| LumB | 204 | 1.663 (1.088-2.541) | **0.019** |  | 1.121 (0.677-1.858) | 0.657 |
| Her2 | 82 | 2.261 (1.325-3.859) | **0.003** |  | 1.377 (0.607-3.121) | 0.444 |
| Basal | 195 | 1.285 (0.833-1.981) | 0.257 |  | 1.210 (0.543-2.693) | 0.641 |
| Anatomic neoplasm subdivisions | 1082 |  |  |  |  |  |
| Left | 563 | Reference |  |  |  |  |
| Right | 519 | 0.766 (0.554-1.057) | 0.105 |  |  |  |
| Risk score | 1082 |  |  |  |  |  |
| Low | 541 | Reference |  |  |  |  |
| High | 541 | 1.618 (1.173-2.233) | **0.003** |  | 1.630 (1.099-2.417) | **0.015** |
